# Influenza vaccine response in community-dwelling German prefrail and frail individuals

**DOI:** 10.1186/s12979-017-0098-z

**Published:** 2017-07-06

**Authors:** Jürgen M. Bauer, Antonio De Castro, Nabil Bosco, Celine Romagny, Rebecca Diekmann, Jalil Benyacoub, Karine Vidal

**Affiliations:** 1 Department of Geriatric Medicine, Carl von Ossietzky University of Oldenburg, Oldenburg, Germany; 2 Nestlé Research Center, Route du Jorat 57, 1000 Lausanne, Switzerland; 3 Present address: Center for Geriatric Medicine, University of Heidelberg and Agaplesion Bethanien Hospital, Heidelberg, Germany; 4 Present address: Nestlé Research Center Asia, 21 Biopolis Road, Singapore, 138567 Singapore

**Keywords:** Elderly, Frailty, Influenza, Vaccination

## Abstract

**Background:**

The age-related dysregulation of the immune system in older persons results in reduced responses to vaccination and greater susceptibility to infection, especially in frail individuals who suffer the greatest of morbidity and mortality due to infection. Recently, significantly reduced anti-influenza antibody titers and increased rates of influenza infection after vaccination were reported in community-dwelling American frail older adults. The aim of our study was to further assess the relative impact of frailty and of each individual Fried frailty criterion on influenza vaccine response. Prefrail and frail community-dwelling German persons aged ≥70 years were recruited for a nutritional randomized double-blind placebo-controlled clinical trial conducted during the 2014–2015 influenza season. Herein, we present a sub-analysis study of the placebo group to compare 76 prefrail and frail participants.

**Results:**

Previous seasonal influenza vaccination rate was relatively high (77.6%) in the 76 volunteers aged from 70 to 93 years. Of these participants, 65.8% were diagnosed as prefrail and 34.2% as frail according to the Fried frailty criteria. In both prefrail and frail groups, elevated levels of pre-vaccination seroprotection were observed to all vaccine strains (H1N1: 54% and 32%, H3N2: 60% and 72%, B: 10% and 16%). Post-vaccination, similar increases in haemagglutination-inhibiting antibody titers were observed for the three vaccine strains in both prefrail and frail groups. No significant difference in geometric mean titer (GMT) ratios and in rates of seroconversion or seroprotection were observed between prefrail and frail groups. Regarding the five Fried frailty criteria, only participants with low physical activity had significantly lower GMT to the strains H3N2 (55.4 vs 103.7, *p* = 0.001) and B (13.9 vs 20.0, *p* = 0.06), as compared to those having normal physical activity.

**Conclusions:**

Influenza vaccine response was not significantly affected by the frail phenotype, as defined by Fried frailty criteria, in community-dwelling German individuals. However, low physical activity may be a relevant predictor of lower serological response in vaccinated older individuals.

**Trial registration:**

Clinicaltrials.gov NCT02262091 (October 8, 2013).

## Background

The flu is a contagious respiratory illness caused by influenza viruses that infects the nose, throat, and lungs causing mild to severe illness. Seasonal influenza still causes significant morbidity and mortality in older adults [1, 2]. The best preventive strategy generally accepted worldwide is a yearly influenza vaccination [3]. However, this preventive strategy is not equally effective across the lifespan and therefore it is still debated in older adults beyond age 65 [4–6]. A large scale retrospective review of influenza vaccine studies performed by Goodwin et al. concluded that influenza-specific antibody responses were reduced in older compared to young adults [7]. However, Mosterin Hopping et al. suggest that the age-associated decline in antibody responses may be an effect of repeated annual influenza vaccination rather than age [8]. In addition to age, multiple chronic conditions, age-related gradual functional decline of the immune system (termed immunosenescence) and frailty may also contribute to the risk of influenza [9–11]. Diminished specific antibody responses observed in older persons may increase the risk of infections and thereby limit the effectiveness of vaccines (i.e. percentage reduction of disease in a vaccinated group of people compared to an unvaccinated group) with fluctuating rates which can drop from 50% to 11% depending on the clinical settings, the vaccine formulation and the circulating virus strains [12, 13]. Hence, despite the promotion of preventive measures including influenza vaccination, a typical influenza season usually results in a significant health care burden and in thousands of deaths particularly in vulnerable older persons. Between 1976 and 2007, an average of 23′000 influenza-associated deaths per year have been reported in United States in persons with underlying respiratory and circulatory causes, with 89% of fatal cases in adults over age 65 [2]. Therefore, influenza associated morbidity and mortality remain a major public health challenge in western societies and they may further increase as a consequence of the demographic change we are facing with the population above 60 expected to double in size by 2050 [14].

Noteworthy, the older population is heterogeneous in terms of its overall health condition comprising psycho-social and medical dimensions which may interfere with their immune competence and vaccine response. This heterogeneity complicates efforts that aim at the improvement of preventive strategies while the latter should be adapted specifically to the needs of sub-populations that are at risk of vaccination failure. Recently the presence or absence of frailty was proposed as a predictor of vaccine response which would be superior to chronological age as the frailty concept reflects the overall functional status of an older individual [15, 16]. Frailty is a geriatric syndrome characterized by a cumulative decline in physiological functions that causes an increased vulnerability to internal and external stressors, e.g. infections [17]. Two diagnostic tools have gained wide recognition among scientists and physicians so far, the first being the Frailty Index of Rockwood et al. [18] and the second being the frailty criteria developed by Fried et al. [19]. Interestingly, Ridda et al. showed that the Frailty Index was a good predictor of the immune response to pneumococcal vaccine in hospitalized older persons [15]. Then, Yao et al. reported similar results in community-dwelling older adults during the 2007–2008 season for influenza vaccination applying the Fried frailty criteria for the first time [16]. Moreover, it is also known that from year to year vaccine effectiveness as measured based on laboratory confirmed influenza cases in a randomized influenza vaccine trial, may fluctuate particularly in older persons. This variability in vaccine effectiveness when comparing different influenza seasons, is largely attributable to the circulating influenza subtype. It is frequently reported that vaccine effectiveness reaches lowest level for influenza A subtype H3N2 than influenza B subtype as shown in the last interim report estimates of 2016–17 seasonal influenza vaccine effectiveness from United States Centers for Disease Control and Prevention [20]. We believe that additional studies are needed to validate the diagnosis of frailty and the intermediate state of pre-frailty with regard to its predictive power of vaccine response. The aim of the present study was to assess the relevance of frailty status based on the Fried frailty criteria on influenza vaccine antibody response during the 2014–2015 seasonal flu vaccine campaign in community-dwelling German older persons. Importantly, we also studied the relevance of individual frailty criteria on each virus subtype antibody titer.

## Results

### Characteristics of the study participants

Demographic and key baseline characteristics of the participants are shown in Table 1. The mean age was 76.0 ± 5.1 years (range, 70–93 years), 46 were females and 30 males, 34 (44.7%) lived alone, 41 (53.9%) as couples and 6 (7.9%) in a nursing home. Among these 76 subjects, 50 (65.8%) were diagnosed as prefrail and 26 (34.2%) as frail based on criteria provided by Fried and colleagues [19]. When analyzing the prevalence of individual frailty criteria, the most prevalent frailty criterion among prefrail study participants was “feeling of exhaustion” (66.7%), followed by “low grip strength” (41.7%) and “low walking speed” (35.4%), while in the frail participants, “feeling of exhaustion” (92.0%) was the most prevalent criterion flowed by “slow walking speed” (80.0%) and “low grip strength” (76.0%). According the MNA, none of the participants was identified as malnourished. At risk for becoming malnourished were similar between prefrail and frail groups (12.2% vs. 11.5%). Blood albumin levels were within the normal range in both prefrail and frail groups. No significant difference in high sensitive CRP was observed between prefrail and frail groups (*p* = 0.139). The frail participants differed significantly from the prefrail participants with regard to quality of life with lower scores on the visual analogue scale (EQ-VAS) (68 ± 15 vs. 80 ± 14; *p* = 0.002) and lower MMSE score (23 ± 2 vs. 28 ± 3; *p* < 0.001). Prior seasonal influenza vaccination rate was similar between prefrail and frail groups (77.1% vs. 76.0%).

**Table 1:** Baseline characterisitcs of study participants

		Prefrail (*n* = 50)	Frail (*n* = 26)
Age (years)	Mean (SD)	74.9 (4.4)	78.1 (5.8)*
Ethnicity (white)	n (%)	50 (100)	26 (100)
Gender (female)	n (%)	30 (60.0)	16 (61.5)
Weight (kg)	Mean (SD)	75.4 (15.5)	79.2 (13.5)
Body Mass Index (kg/m^2^)	Mean (SD)	26.5 (3.6)	28.9 (4.2)*
Diastolic Blood Pressure (mmHg)	Mean (SD)	76.6 (7.9)	79.0 (11.4)
Systolic Blood Pressure (mmHg)	Mean (SD)	139.3 (16.9)	145.9 (19.6)
Pulse rate (bpm)	Mean (SD)	71.7 (9.8)	72 (11.9)
MMSE score (/30)^a^	Mean (SD)	28.0 (3.0)	23 (2.0)***
MNA SF score (/14)^b^	Mean (SD)	13.0 (1.0)	13 (1.0)
Good nutritrional status	n (%)	43 (87.8)	23 (88.5)
Risk of malnutition	n (%)	6 (12.2)	3 (11.5)
Malnourished	n (%)	0 (0)	0 (0)
IADL score (/8)^c^	Mean (SD)	7.0 (1.0)	7.0 (2.0)
EQ-5D dimension (no problem)^df^			
Mobility	n (%)	34 (70.8)	7 (28.0)
Self Care	n (%)	47 (97.9)	18 (72.0)
Usual Activity	n (%)	38 (79.2)	13 (52.0)
Pain/Discomfort	n (%)	28 (58.3)	6 (24.0)
Anxiety/Depression	n (%)	38 (79.2)	20 (80.0)
EQ-VAS score^e^	Mean (SD)	80.0 (14.0)	68.0 (15.0)***
Frailty criteria^f^			
Feeling of exhaustion	n (%)	32 (66.7)	23 (92.0)
Low grip strength	n (%)	20 (41.7)	19 (76.0)
Low physical activity	n (%)	4 (8.3)	13 (52.0)
Low walking speed	n (%)	17 (35.4)	20 (80.0)
Involuntary recent weight Loss^f^	n (%)	5 (10.4)	5 (20.0)
Domestic status^f^			
Alone	n (%)	17 (34.0)	17 (65.4)
In couple	n (%)	32 (64.0)	9 (34.6)
Dependent	n (%)	2 (4.0)	4 (15.4)
Tobacco use			
Never	n (%)	31 (62.0)	14 (53.8)
Former	n (%)	14 (28.0)	11 (42.3)
Current	n (%)	5 (10.0)	1 (3.8)
Alcohol consumption			
Never	n (%)	19 (38.0)	12 (46.2)
Former	n (%)	4 (8.0)	3 (11.5)
Current	n (%)	27 (54.0)	11 (42.3)
Blood biochemistry			
AST (U/L)	Mean (SD)	25.4 (7.1)	22.0 (5.9) *
Albumin (g/dl)	Mean (SD)	4.2 (0.3)	4.2 (0.3)
Total Protein (g/L)	Mean (SD)	69.0 (4.1)	67.9 (3.7)
Creatinin (mg/dl)	Mean (SD)	0.9 (0.3)	0.9 (0.3)
Glucose (mg/dl)	Mean (SD)	101.1 (23.3)	105.5 (20.8)
Hemoglobin (g/dL)	Mean (SD)	14.0 (1.3)	14.0 (1.0)
Hematocrit (%)	Mean (SD)	42.5 (3.2)	42.8 (3.1)
IgG (g/L)	Mean (SD)	10.5 (2.1)	10.2 (1.6)
Hs-CRP (mg/l)	Mean (SD)	2.8 (2.5)	5.3 (8.0)
Hematology			
Platelets (%)	Mean (SD)	240.9 (55.5)	241.0 (58.7)
Erythrocytes (10^E^12/L)	Mean (SD)	4.5 (0.4)	4.6 (0.3)
Basophil (%)	Mean (SD)	0.6 (0.3)	0.5 (0.2)
Eosinophil (%)	Mean (SD)	3.7 (2.2)	3.1 (2.4)
Neutrophils (%)	Mean (SD)	57.8 (8.9)	60.4 (10.8)
Monocytes (%)	Mean (SD)	7.9 (2.0)	8.3 (2.6)
Lymphocytes (%)	Mean (SD)	28.8 (8.2)	26.9 (8.7)
Leucocytes(10E9/L)	Mean (SD)	6.2 (1.8)	6.4 (1.6)
Flu vaccinated previous year^f^	n (%)	37 (77.1)	19 (76.0)

^a^Mini Mental State Examination. Score > 24 = normal cognition, 19–23 = mild cognitive impairment, 10–18 = moderate and <9 = severe. ^b^Mini Nutritional Assessment Short Form. Score from 12 to 14 = good nutritional status, 8–11 = at risk of manutrition and 0–7 = malnourished.﻿ ^c^Instrumental Activities of Daily Living. Score from 0 = low (person very dependent) to 8 = high (person independent).﻿ ^d^EQ-5D: EuroQoL 5 dimensions. Data correspond to the health state description “Having no problems” in the following 5 dimensions: Mobility = walking ability, Self-care dimension = ability to wash or dress by oneself, Usual activities = performance in work, study, housework, family or leisure activities, Pain/Discomfort = pain or discomfort, Anxiety/Depression = anxiety or depression.﻿ ^e^EQ-VAS: Visual Analogue Scale. Self evaluation of health status on the day of the interview. Score from 0 = low (worst imaginable health state) to 100 = high (best imaginable health state). ^f^Refers to data with Prefrail (*n* = 48) and Frail (*n* = 25). *, *p*﻿ < 0.05; ***, *p* < 0.001

### Seasonal influenza vaccine response in all participants

Vaccination resulted in significant increases in HAI antibodies to the three subtypes of the influenza vaccine (H1N1, H3N2 and B) in the study participants comparing pre and post-vaccination antibody titers (*p* < 0.001 for all subtypes) (Table 2). Pre-vaccination and post-vaccination GMT against both H1N1 and H3N2 subtypes of influenza A were higher than GMT against the virus type B. The GMT ratios obtained for the H1N1 and H3N2 were above 2, and the GMT ratio for the B virus type was barely equal to 2 (Table 2). Seroconversion rates were quite modest for both A and B types (47.9% for H1N1, 31.3% for H3N2 and 16.7% for the B) (Table 3). Baseline seroprotection rates to the influenza strains were relatively high with 46.6% for H1N1, 64.4% for H3N2 and 12.3% for the B type (Table 4). After vaccination, seroprotection rates were increased up to 84.9% for H1N1 and 89.0% for H3N2, and only up to 31.5% for the B type.

**Table 2:** Geometric mean titers in prefrail and frail subjects, and all study subjects before and 4 weeks after influenza vaccination

Vaccine		Prefrail (*n* = 48)				Frail (*n* = 25)				All (*n* = 73)				*p* value (prefrail vs. frail)
		GMT	GMSD	Lower	Upper	GMT	GMSD	Lower	Upper	GMT	GMSD	Lower	Upper	
A/California/7/2009 (H1N1)	Before	27.9	3.7	7.6	102.4	17.9	3.0	6.0	53.7	24.0	3.3	7.2	79.7	
	After	89.8^a^	3.3	27.0	298.1	84.6^a^	4.5	18.8	380.7	88.0^a^	3.7	24.0	322.9	
	Ratio	3.2				4.7				3.9				0.57
A/Texas/50/2012 (H3N2)	Before	37.2	2.7	13.7	101.1	42.3	2.2	19.0	94.1	38.9	2.7	14.3	105.7	
	After	92.4^a^	3.3	27.8	306.8	84.6^a^	2.5	34.4	208.1	89.7^a^	3.0	29.9	269.5	
	Ratio	2.5				2.0				2.3				0.38
B/Massachusetts/2/2012	Before	9.0	2.0	4.5	18.1	9.2	2.2	4.1	20.5	9.1	2.0	4.5	18.3	
	After	17.1^a^	2.7	6.3	46.5	21.1^a^	3.3	6.4	70.1	18.4^a^	3.0	6.1	55.3	
	Ratio	1.9				2.3				2.0				0.38

GMT represents geometric mean for the titer counts and GMSD refers to it’s corresponding standard deviation. The calculation of the confidence interval is by dividing the GMT by the GMSD for the lower bound and multiplying it for the upper bound. "Before" refers to baseline samples (i.e. 4 weeks before vaccination) and "After" refers to 4 weeks after vaccination. ^a^Pre-vaccination GMT significantly different from post-vaccination GMT (*p* < 0.01)﻿. The *P*-values presented here pertain to the comparison of ratios between the prefrail and frail groups. A linear mixed model was used and a logarithmic transformation was applied to the titer counts (before and after) and their difference would result in the before and after ratio. Adjustment for age and BMI were done (as they were found to be significant at baseline demographics level) but no significant difference was observed from the simpler model. Therefore the results presented here are coming from the simpler model

**Table 3:** Seroconversion in prefrail and frail subjects, and in all study subjects 4 weeks after influenza vaccination

Vaccine strain	Prefrail (*n* = 48) n/N (%)	Frail (*n* = 25) n/N (%)	All (*n* = 73) n/N (%)	*p* value (prefrail vs. frail)
A/California/7/2009 (H1N1)	23/48 (47.9)	13/25 (52.0)	36/73 (49.3)	0.93
A/Texas/50/2012 (H3N2)	15/48 (31.3)	7/25 (28.0)	22/73 (30.1)	0.99
B/Massachusetts/2/2012	8/48 (16.7)	4/25 (16.0)	12/73 (16.4)	1.00

Data are numbers of seroconverted (n)/total (N) and % of seroconverted subjects in parentheses

**Table 4:** Seroprotection in prefrail and frail subjects, and in all study subjects before and 4 weeks after influenza vaccination

Vaccine strain		Prefrail (*n* = 48) n/N (%)	Frail (*n* = 25) n/N (%)	All (*n* = 73) n/N (%)	*p* value (prefrail vs. frail)
A/California/7/2009 (H1N1)	Before	26/48 (54.2)	8/25 (32.0)	34/73 (46.6)	0.12
	After	42/48 (87.5)	20/25 (80.0)	62/73 (84.9)	0.61
A/Texas/50/2012 (H3N2)	Before	29/48 (60.4)	18/25 (72.0)	47/73 (64.4)	0.47
	After	43/48 (89.6)	22/25 (88.0)	65/73 (89.0)	1.00
B/Massachusetts/2/2012	Before	5/48 (10.4)	4/25 (16.0)	9/73 (12.3)	0.75
	After	15/48 (31.3)	8/25 (32.0)	23/73 (31.5)	1.00

Data are numbers of seroprotected (n)/total (N) and % of seroprotected subjects in parentheses; “Before” refers to baseline samples (i.e. 4 weeks before vaccination) and “After” to 4 weeks after vaccination

### Effect of frailty on influenza vaccine response

There were no statistically significant differences between prefrail and frail groups with regard to baseline GMT against any of the vaccine viral strains (Table 2). Following influenza vaccination, both prefrail and frail groups had significant increases in HAI antibodies against the three vaccine strains representing H1N1, H3N2 and B viruses comparing pre and post-vaccination antibody titers (*p* < 0.001 or 0.01 for prefrail or frail respectively for all subtypes). However, there were no statistically significant differences between prefrail and frail groups with regard to post−/pre-vaccination GMT ratios (Table 2) and seroconversion rates (Table 3) for any of the vaccine viral strains. There were elevated levels of pre-vaccination seroprotection (i.e. HAI titers ≥40) against all vaccine subtypes in both prefrail and frail groups (H1N1: 54.2% and 32.0%, H3N2: 60.4% and 72.0%, B: 10.4% and 16.0% in prefrail and frail participants, respectively) (Table 4). Only a trend was observed for a lower level of seroprotection at baseline against the H1N1 subtype in the frail group as compared to the prefrail group (32.0% vs. 54.2%; *p* = 0.12). After vaccination, seroprotection rates increased to a similar degree in prefrail and frail groups (H1N1: 87.5% vs. 80.0%, H3N2: 89.6% vs. 88.0%, B: 31.3% vs. 32.0% in prefrail vs. frail, respectively) (Table 4). Overall, we could not find a statistically significant effect of the frailty status on influenza vaccine response in this community-dwelling individuals that participated in the present study.

### Influenza vaccine response according to individual frailty criteria

To assess whether individual frailty criteria interfere with the vaccine response in older individuals, GMT at baseline and after vaccination were evaluated for the five frailty criteria individually (Table 5). At baseline, there were no statistically significant differences in GMT against the three vaccine viral strains for any of the five frailty criteria. After vaccination, there were no statistically significant differences in GMT for all three vaccine viral strains between the subjects who were positive or negative for “low grip strength” and “exhaustion”. However, subjects scoring positive for the criterion “low walking speed” had a trend for lower GMT to H1N1 when compared to those who were negative for this criterion (72.8 vs. 106.8; *p* = 0.07), while having significantly higher GMT to H3N2 strain (110.0 vs 72.7; *p* = 0.03) and B strain (24.1 vs. 13.9; *p* = 0.01). Subjects who were positive for the “weight loss” criterion had significantly higher GMT to H1N1 as compared to those who were negative for this criterion (121.3 vs. 83.6; *p* = 0.02). Subjects scoring positive for the “low physical activity” criterion had significant lower GMT against the H3N2 and the B strains when compared to those who were negative for this criterion (H3N2: 55.4 vs. 103.7; *p* = 0.001, B: 13.9 vs. 20.0; *p* = 0.06, respectively).

**Table 5:** GMT before and 4 weeks after vaccination according to the frailty criteria

		Frailty criteria									
		Weight loss		Low grip strength		Exhaustion		Low walking speed		Low physical activity	
Vaccine strain		Yes (*n* = 10)	No (*n* = 63)	Yes (*n* = 39)	No (*n* = 34)	Yes (*n* = 55)	No (*n* = 18)	Yes (*n* = 37)	No (*n* = 36)	Yes (*n* = 17)	No (*n* = 56)
A/California/7/2009 (H1N1)	Before	16.2	25.5	23.9	24.0	21.8	31.7	23.7	24.2	20.0	25.3
	After	121.3	83.6^a^	93.9	81.6	81.0	113.1	72.8	106.8^b^	90.4	87.2
A/Texas/50/2012 (H3N2)	Before	30.3	40.4	42.2	35.4	41.0	33.0	41.5	36.3	38.4	39.0
	After	65.0	94.4	93.9	85.0	98.0	68.6	110.0	72.7^c^	55.4	103.7^e^
B/Massachusetts/2/2012	Before	6.6	9.6	8.8	9.4	9.9	7.1	9.6	8.6	9.2	9.1
	After	18.7	18.3	17.3	19.6	20.5	13.1	24.1	13.9^d^	13.9	20.0^f^

Data are geometric mean HAI titers (GMT). “B﻿efore” refers to baseline samples (i.e. 4 weeks before vaccination) and “after” refers to 4 weeks after vaccination. *P* values for difference in GMT between the subjects positive or negative for the individual frailty criteria: ﻿^a^ *p* = 0.02; ﻿^b^ *p* = 0.07; ^c^ *p* = 0.03; ^d^ *p* = 0.01; ^e^ *p* ﻿= 0.0﻿01; ^f﻿^ *p* = 0.06

Summarizing the above, these results suggest that among the five frailty criteria, the “low physical activity” criterion showed the strongest association with a diminished serological response after influenza vaccination in prefrail and frail individuals.

## Discussion

Previous studies have reported that frailty had a significant impact on influenza [16] and pneumococcal vaccine responses [15]. It has even been suggested that the frailty status could be a stronger predictor of vaccine response than age [16]. In the present study, our data have not provided evidence for a weaker antibody response after influenza vaccination in frail individuals when compared to prefrail individuals. Nevertheless, our study corroborates recent results drawn from secondary outcomes of larger vaccine studies where the frailty criteria of Rockwood were slightly adapted and applied [21, 22]. In a large-scale efficacy and immunogenicity trial of standard-dose versus high-dose influenza vaccines DiazGranados et al. did not find an interaction between frailty status and HAI vaccine antibody titers [21]. In addition, the frailty status did not influence the incidence of influenza-like illnesses in older adults above 65 years of age [21]. Talbot et al. analyzed whether frailty status confounds influenza vaccine effectiveness estimates in a so-called case positive test-negative study design [22]. Adults above 50 years of age hospitalized with respiratory symptoms between 2006 and 2012 where tested for influenza, assessed for frailty and asked for their vaccination history. In this study despite a higher prevalence of hospitalizations for respiratory symptoms in prefrail and frail individuals, influenza vaccine effectiveness estimates were not significantly different between frail and non-frail older persons. The authors concluded that frailty was not a significant confounder in vaccine effectiveness studies. Neither the present study that applied the Fried frailty criteria nor previous studies using the Frailty Index of Rockwood found a significant impact of the frailty syndrome on vaccine response based on measures of antibody titers and effectiveness based on incidence of influenza. Nevertheless, limitations in the study by Talbot et al. were discussed recently with regard to vaccine effectiveness estimates [22]. Indeed, the applied strategy for statistical analysis was challenged based on the existing bias that frailty can still influence decision to vaccinate as well as risk of hospitalization and death from influenza [23].

Our results also highlight the difficulty to discriminate immune competence between prefrail and frail individuals. Indeed, differentiating robust (no parameter), prefrail (1–2 parameters) and frail (3–5 parameters) persons may require additional standardization and possibly alternative approaches. Although the diagnosis of pre-frailty and frailty were not associated with vaccine response standard readouts as HAI titers, rate of seroconversion and seroprotection, we further analyzed potential associations with individual frailty criteria by exploring the immunogenicity of the influenza vaccine according to the participants’ baseline individual frailty criteria: weakness, slowness, low level of physical activity, self-reported exhaustion, and unintentional weight loss. We could show differences for associations of the different Fried criteria with the vaccine response. Our analysis suggests that among the five parameters used to diagnose frailty, low physical activity may be regarded as a relevant predictor of a weak humoral response to seasonal influenza vaccination in prefrail and frail individuals particularly for influenza B type and A (H3N2) subtype. Variability in vaccine effectiveness when comparing different influenza seasons is linked to the circulating influenza subtype. During H3N2 endemic winter seasons an increase in all-cause mortality is often observed. Viral epidemiology studies suggest that circulation of influenza virus, in particular with the virus type A subtype H3N2, is the main seasonal driver of excess mortality among the elderly [24]. This has been shown in the United States [25] and recently confirmed in Europe [26]. Our observation would suggest that lack of exercise can be consider as a risk factor in elderly during H3N2 endemic winter season.

We know that exercise is a powerful preventive strategy for several aspects of health in older adults including the immune system (for review [27]). However, most of the knowledge in this field has been established for younger adults and athletes. It may also be suggested that older adults may benefit from physical exercise programs. Indeed, beyond general health improvement and reduction of frailty status, currently recommended exercise programs may also help to improve vaccine immunogenicity and reduce infection rates in older persons. In that line, a recent work of de Araujo et al. showed that exercise promotes strong and long-lasting immune responses to influenza vaccine in older persons [28].

We should acknowledge some important limitations to our study. It is worth notice that in many studies, an important confounder like nutritional status was not systematically assessed and this may explain different outcomes. In our study, malnutrition was absent according to the MNA. It may be suggested that we enrolled a population that may be more immunocompetent than the prefrail and frail populations that were included in other studies. Moreover, it should also be noticed that the participants of the present study were drawn from regions with high vaccination coverage. This may be relevant as a recent report from Mosterin Hopping et al. showed that numbers of vaccinations and age are confounders of vaccination effectiveness, suggesting that repetitive vaccinations mask the differences in influenza vaccine responses observed in aged population compared to younger adults [8]. Of note, about 77% of our study participants had received a seasonal influenza vaccination the year prior to our trial. Also, due to their age range, it is reasonable to assume that they had multiple seasonal influenza shots and/or exposure to various influenza strains over the years prior to the current study. Indeed, the participants had relatively high pre-vaccination antibody titers and even seroprotection rates to the influenza vaccine strains, especially to the H1N1 and H3N2 strains. The respective percentages were very similar to the results reported in other studies with healthy older robust volunteers [7]. These characteristics of our study participants may thus be regarded as a limitation of our study. To clarify this issue a study may be considered with robust, prefrail and frail participants who present with a naïve vaccination status. However, such a study may not be seen as realistic for Western European populations, nor ethical.

Our study included individuals with a well-defined diagnosis of frailty based on the Fried frailty criteria and the participants were community-dwelling older individuals. Classical phenotypical characteristics of frail subjects like reduction in quality of life, cognitive and mobility scores were observed as compared to prefrail subjects and can be attributed to age (with a mean of 3 year old difference) instead of frailty per se. However, it did not impact the vaccine response in our study. Also, we do not exclude that institutionalized elderly may behave differently regarding frailty status and vaccine response at the same age.

Unfortunately, we could not compare our results in prefrail and frail older individuals to results obtained in non-frail (i.e. robust) older adults as only individuals diagnosed as prefrail or frail were included in the present study which represent a sub-analysis of the placebo arm of an intervention trial. It is worth noticing that although Yao et al. observed some differences in influenza vaccine response between prefrail and frail individuals, the more consistent and significant changes were reported when the frail versus robust study participants were compared [16]. Herein, frailty according to the Fried frailty criteria was overall not associated with reduction in vaccine response in community dwelling older persons. Thus, our results do not support the use of these criteria as a whole to predict influenza vaccine response.

## Conclusion

In conclusion, we showed that prefrail and frail individuals have a preserved humoral immunity, respond normally and equally-well to the vaccine, which argues towards the importance of influenza vaccination in the older population in general.

## Methods

### Study design, ethics and trial registration

The dataset analyzed in the present article focuses exclusively on the participants who received the placebo treatment in a multi-center, prospective, randomized, double-blind, placebo-controlled, parallel clinical trial conducted in prefrail and frail older people ≥70 years of age in Germany. The full data set will be documented separately. The original trial was designed to demonstrate that, relative to placebo, supplementation with an investigational compound would improve immune response in prefrail/frail older persons. The study protocol was approved by the following German Ethics Committees: Muenster (Muenster), Saarland (Saarbruecken), Medical Chamber of Nordrhein (Duesseldorf), State Medical Chamber of Baden (Stuttgart), Geschäftsstelle (Berlin), and Ärztekammer Niedersachsen (Hannover). The trial was registered on clinicaltrials.gov (Identifier: NCT02262091) and conducted according to the guidelines laid down in the Declaration of Helsinki. Written informed consent was obtained from all study participants after the nature and possible consequences of the studies had been fully explained and before they were screened for eligibility criteria.

### Study participants

Prior to the influenza season of 2014–2015, community-dwelling older volunteers ≥70 years were recruited through adverts in print media and the internet, flyers in GPs offices and by word of mouth with their GP. Inclusion criteria were: prefrail and frail subjects, as determined by the Fried frailty criteria, who were 70 years and older, willing to get a seasonal influenza vaccination and who did not meet any of the exclusion criteria. Exclusion criteria included: rapid deteriorating health status, including terminal, severe or uncontrolled acute as well as chronic diseases (e.g. diabetes, carcinoma, renal diseases), allergy to eggs or influenza vaccine components, any vaccination in the last 3 months, current use of immune modulating medication (including use of steroids, immune suppressive treatment), use of antibiotics within last 2 months, regular consumption of prebiotics or probiotics, yogurts or other dietary supplements, blood transfusion or donation within the last 4 weeks, family history of Guillain-Barre syndrome, body mass index (BMI) > 35 kg/m^2^, likelihood to be non-compliant with study procedure, current participation or participation in another clinical trial in the previous 4 weeks. Screening also included a medical history (co-morbidities, allergies, medication, and influenza vaccination during the previous year), clinical examination (anthropometric measures, blood pressure, pulse rate), geriatric questionnaires (Mini-mental state examination: MMSE, short form of the mini nutritional assessment: MNA, instrumental activities of daily living: IADL, and quality of life questionnaire: EQ-5D-5L™) and routine laboratory tests (blood chemistry and hematology). Baseline characteristics were collected at enrollment.

### Fried frailty criteria

Prefrail and frail older people were diagnosed according to the five criteria developed by Fried and colleagues [19], i.e. weight loss, exhaustion, grip strength, walk time and physical activity. The cut-offs values applied for each criteria were the same as in the original study that defined the frailty phenotype [19]. Participants were diagnosed as frail if three or more of these five criteria were assessed as positive and those with one or two positive criteria were considered to be prefrail [19].

### Influenza vaccination and measurement of specific antibody response

Subjects meeting eligibility criteria received a standard dose of a seasonal, trivalent, inactivated, split-virus influenza vaccine (Influsplit SSW® 2014–2015, Lot N°:AFLUA833CA) administrated by intramuscular injection according to the manufacturer’s recommendations at day 30 after the baseline visit. The 2014–2015 influenza seasonal vaccine contained 15 mcg of hemagglutinin of each of the following viral strains: A/California/7/2009 (H1N1), A/Texas/50/2012 (H3N2) and B/Massachusetts/2/2012. Blood samples were collected at the baseline visit (V0, day 0), at visit 1 (day 30) prior to administration of the vaccine, and at visit 2 (day 60). Sera were stored at −80 °C and sent on dry ice to the Pasteur Institute (Paris, France) for blinded analysis of vaccine-specific antibody titer measurements. Serum antibody titers against the three viral strains of the vaccine were measured by hemagglutination inhibition (HAI) assay with guinea pig red blood cells, according to standard protocols (Manuel for the laboratory diagnosis and virological surveillance of influenza, World Health Organization 2011). Titers which were <10 (below the detection limit) were expressed as 5, to represent half of the detection threshold. Three standard measures of vaccine response were studied for each vaccine strain: 1) geometric mean titer (GMT) of HAI antibodies, 2) seroconversion rate defined as percentage of subjects with an increase from <1:10 to ≥1:40 or a ≥ 4-fold increase in HAI titers, 3) seroprotection rate defined as percentage of subjects with HAI titers ≥40.

### Statistical analysis

Data for demographic characteristics were expressed as means (± SD) for continuous variables and counts (%) for categorical variables. Three demographic data were shown to have statistically significant differences between the frail and prefrail subgroups; age, BMI and overall quality of life score. Change from baseline of HAI titer data were evaluated with the use of a linear model taking as fixed effects the baseline titer value and the frailty criteria. Logarithmic transformation was applied on the HAI titer data to produce the following model: *log(HAIafter/HAIbefore) = Intercept +* *β*_*1*_ *log(HAIbefore) +* *β*_*2*_ *Frailty Status +* *ε,* where *HAI*_*after*_ refers to the titer count after vaccination and *HAI*_*before*_ refers to the titer count before vaccination. The intercept represents a general mean with the prefrail group as a reference and *β*_*1*_ pertains to the coefficient of how baseline titer count impacts the dependent variable and *β*_*2*_ on how the Frail group differs from the Prefrail group. The term *log*(*HAIafter/HAIbefore*) is equivalent to *log(HAI*_*after*_ *)* – *log(HAI*_*before*_ *)* via mathematical property of the logarithmic function. Seroprotection and seroconversion rates were analyzed using Fisher’s exact test since number of incidences in most cases are lower than 10. Both SAS ver. 9.3 and R ver. 3.2.3 were used for the statistical analyses. The subgroup of subjects belonging to the placebo group was big enough to detect a 0.7 or higher effect size (a difference in GMT ratio of 70% of the standard deviation between the frail and prefrail) with the conventional 80% power. No adjustment for multiplicity (adjustment of the type-I error; α) was applied to the statistical analyses mentioned above due to the exploratory nature of this subgroup analysis.

## Abbreviations

GMT: Geometric mean titers
HAI: Hemagglutination inhibition
IADL: Instrumental activities of daily living
MMSE: Mini mental state examination
MNA: Mini nutritional assessment
QOL: Quality of life

## References

[1] Nicholson KG, Wood JM, Zambon M (2003). Influenza. Lancet.

[2] Centers for Disease Control and Prevention. Estimates of Deaths Associated with Seasonal Influenza — United States, 1976–2007. MMWR 2010; 59(33):1057–62. 20798667

[3] Monto AS (2010). Seasonal influenza and vaccination coverage. Vaccine.

[4] Jefferson T, Di Pietrantonj C, Al-Ansary LA, Ferroni E, Thorning S, Thomas RE (2010). Vaccines for preventing influenza in the elderly. Cochrane Database Syst Rev.

[5] Michiels B, Govaerts F, Remmen R, Vermeire E, Coenen S (2011). A systematic review of the evidence on the effectiveness and risks of inactivated influenza vaccines in different target groups. Vaccine.

[6] Osterholm MT, Kelley NS, Sommer A, Belongia EA (2012). Efficacy and effectiveness of influenza vaccines: a systematic review and meta-analysis. Lancet Infect Dis.

[7] Goodwin K, Viboud C, Simonsen L (2006). Antibody response to influenza vaccination in the elderly: a quantitative review. Vaccine.

[8] Mosterín Höpping A, McElhaney J, Fonville JM, Powers DC, Beyer WE, Smith DJ (2016). The confounded effects of age and exposure history in response to influenza vaccination. Vaccine.

[9] Pawelec G, Larbi A, Derhovanessian E (2010). Senescence of the human immune system. J Comp Pathol.

[10] Palmer DB (2013). The effect of age on thymic function. Front Immunol.

[11] Pera A, Campos C, López N, Hassouneh F, Alonso C, Tarazona R, Solana R (2015). Immunosenescence: implications for response to infection and vaccination in older people. Maturitas.

[12] Govaert TM, Thijs CT, Masurel N, Sprenger MJ, Dinant GJ, Knottnerus JA (1994). The efficacy of influenza vaccination in elderly individuals. A randomized double-blind placebo-controlled trial. JAMA.

[13] McLean HQ, Thompson MG, Sundaram ME, Kieke BA, Gaglani M, Murthy K, Piedra PA, Zimmerman RK, Nowalk MP, Raviotta JM, Jackson ML, Jackson L, Ohmit SE, Petrie JG, Monto AS, Meece JK, Thaker SN, Clippard JR, Spencer SM, Fry AM, Belongia EA (2015). Influenza vaccine effectiveness in the United States during 2012-2013: variable protection by age and virus type. J Infect Dis.

[14] United Nations, Department of Economic and Social Affairs, Population Division (2015). World Population Ageing 2015 (ST/ESA/SER.A/390).

[15] Ridda I, Macintyre CR, Lindley R, Gao Z, Sullivan JS, Yuan FF, McIntyre PB (2009). Immunological responses to pneumococcal vaccine in frail older people. Vaccine.

[16] Yao X, Hamilton RG, Weng NP, Xue QL, Bream JH, Li H, Tian J, Yeh SH, Resnick B, Xu X, Walston J, Fried LP, Leng SX (2011). Frailty is associated with impairment of vaccine-induced antibody response and increase in post-vaccination influenza infection in community-dwelling older adults. Vaccine.

[17] Morley JE, Vellas B, van Kan GA, Anker SD, Bauer JM, Bernabei R, Cesari M, Chumlea WC, Doehner W, Evans J, Fried LP, Guralnik JM, Katz PR, Malmstrom TK, McCarter RJ, Gutierrez Robledo LM, Rockwood K, von Haehling S, Vandewoude MF, Walston J (2013). Frailty consensus: a call to action. J Am Med Dir Assoc.

[18] Mitnitski AB, Graham JE, Mogilner AJ, Rockwood K (2002). Frailty, fitness and late-life mortality in relation to chronological and biological age. BMC Geriatr.

[19] Fried LP, Tangen CM, Walston J, Newman AB, Hirsch C, Gottdiener J, Seeman T, Tracy R, Kop WJ, Burke G, McBurnie MA; Cardiovascular Health Study Collaborative Research Group Frailty in older adults: evidence for a phenotype. J Gerontol A Biol Sci Med Sci 2001; 56(3):M146-M156. 10.1093/gerona/56.3.m146 11253156

[20] Flannery B, Chung JR, Thaker SN, Monto AS, Martin ET, Belongia EA, McLean HQ, Gaglani M, Murthy K, Zimmerman RK, Nowalk MP, Jackson ML, Jackson LA, Foust A, Sessions W, Berman L, Spencer S, Fry AM (2017). Interim estimates of 2016–17 seasonal influenza vaccine effectiveness — United States, February 2017. MMWR Morb Mortal Wkly Rep.

[21] DiazGranados CA, Dunning AJ, Robertson CA, Talbot HK, Landolfi V, Greenberg DP (2015). Efficacy and immunogenicity of high-dose influenza vaccine in older adults by age, comorbidities, and frailty. Vaccine.

[22] Talbot HK, Nian H, Chen Q, Zhu Y, Edwards KM, Griffin MR (2016). Evaluating the case-positive, control test-negative study design for influenza vaccine effectiveness for the frailty bias. Vaccine.

[23] Sullivan SG, Cowling BJ, Greenland S (2016). Frailty and influenza vaccine effectiveness. Vaccine.

[24] Matias G, Taylor R, Haguinet F, Schuck-Paim C, Lustig R, Shinde V (2017). Estimates of hospitalization attributable to influenza and RSV in the US during 1997-2009, by age and risk status. BMC Public Health.

[25] Thompson WW, Shay DK, Weintraub E, Brammer L, Cox N, Anderson LJ, Fukuda K (2003). Mortality associated with influenza and respiratory syncytial virus in the United States. JAMA.

[26] Vestergaard LS, Nielsen J, Krause TG, Espenhain L, Tersago K, Bustos Sierra N, Denissov G, Innos K, Virtanen MJ, Fouillet A, Lytras T, Paldy A, Bobvos J, Domegan L, O’Donnell J, Scortichini M, de Martino A, England K, Calleja N, Van Asten L, Teirlinck AC, Tønnessen R, White RA, P Silva S, Rodrigues AP, Larrauri A, Leon I, Farah A, Junker C, Sinnathamby M, Pebody RG, Reynolds A, Bishop J, Gross D, Adlhoch C, Penttinen P, Mølbak K (2017). Excess all-cause and influenza-attributable mortality in Europe, December 2016 to February 2017. Euro Surveill.

[27] Simpson RJ, Kunz H, Agha N, Graff R (2015). Exercise and the regulation of immune functions. Prog Mol Biol Transl Sci.

[28] de Araújo AL, Silva LC, Fernandes JR, Matias Mde S, Boas LS, Machado CM, Garcez-Leme LE, Benard G (2015). Elderly men with moderate and intense training lifestyle present sustained higher antibody responses to influenza vaccine. Age.

